# Effects of Live and Peptide-Based Antimicrobiota Vaccines on *Ixodes ricinus* Fitness, Microbiota, and Acquisition of Tick-Borne Pathogens

**DOI:** 10.3390/pathogens14030206

**Published:** 2025-02-20

**Authors:** Apolline Maitre, Lourdes Mateos-Hernandez, Myriam Kratou, Natalia Egri, Jennifer Maye, Manel Juan, Adnan Hodžić, Dasiel Obregón, Lianet Abuin-Denis, Elianne Piloto-Sardinas, Andrea C. Fogaça, Alejandro Cabezas-Cruz

**Affiliations:** 1 Laboratoire de Santé Animale, Anses, INRAE, Ecole Nationale Vétérinaire d’Alfort, UMR BIPAR, 94700 Maisons-Alfort, France; apolline.maitre@orange.fr (A.M.); lourdes.mateos@vet-alfort.fr (L.M.-H.); labuind@gmail.com (L.A.-D.); elianne9409@gmail.com (E.P.-S.); 2 UR 0045 Laboratoire de Recherches Sur Le Développement de L’Elevage (SELMET-LRDE), INRAE, 20250 Corte, France; 3 EA 7310, Laboratoire de Virologie, Université de Corse, 20250 Corte, France; 4 Laboratory of Microbiology, National School of Veterinary Medicine of Sidi Thabet, University of Manouba, Manouba 2010, Tunisia; mariem.kratou@hotmail.com; 5 Servei d’Immunologia, Hospital Clínic de Barcelona, IDIBAPS, Universitat de Barcelona, 08036 Barcelona, Spain; egri@clinic.cat (N.E.); mjuan@clinic.cat (M.J.); 6 SEPPIC Paris La Défense, 92250 La Garenne Colombes, France; jennifer.maye@vet-alfort.fr; 7 Centre for Microbiology and Environmental Systems Science, Department of Microbiology and Ecosystem Science, Division of Microbial Ecology, University of Vienna, 1090 Vienna, Austria; adnan.hodzic@univie.ac.at; 8 School of Environmental Sciences, University of Guelph, 50 Stone Rd E, Guelph, ON N1H 2W1, Canada; dasielogv@gmail.com; 9 Animal Biotechnology Department, Center for Genetic Engineering and Biotechnology, Avenue 31 Between 158 and 190, P.O. Box 6162, Havana 10600, Cuba; 10 Direction of Animal Health, National Center for Animal and Plant Health, Carretera de Tapaste y Autopista Nacional, Apartado Postal 10, San José de las Lajas 32700, Cuba; 11 Department of Parasitology, Institute of Biomedical Sciences, University of São Paulo, São Paulo 05508-000, Brazil

**Keywords:** antimicrobiota vaccines, ticks, *Ixodes ricinus* microbiota, tick-borne disease control, microbial network analysis

## Abstract

This study explored the effects of antimicrobiota vaccines on the acquisition of *Borrelia* and *Rickettsia*, and on the microbiota composition of *Ixodes ricinus* ticks. Using a murine model, we investigated the immunological responses to live *Staphylococcus epidermidis* and multi-antigenic peptide (MAP) vaccines. Immunized mice were infected with either *Borrelia afzelii* or *Rickettsia helvetica*, and subsequently infested with pathogen-free *I. ricinus* nymphs. We monitored the tick feeding behavior, survival rates, and infection levels. Additionally, we employed comprehensive microbiota analyses, including the alpha and beta diversity assessments and microbial co-occurrence network construction. Our results indicate that both live *S. epidermidis* and MAP vaccines elicited significant antibody responses in mice, with notable bactericidal effects against *S. epidermidis*. The vaccination altered the feeding patterns and fitness of the ticks, with the Live vaccine group showing a higher weight and faster feeding time. Microbiota analysis revealed significant shifts in the beta diversity between vaccine groups, with distinct microbial networks and taxa abundances observed. Notably, the MAP vaccine group exhibited a more robust and complex network structure, while the Live vaccine group demonstrated resilience to microbial perturbations. However, the effects of antimicrobiota vaccination on *Borrelia* acquisition appeared taxon-dependent, as inferred from our results and previous findings on microbiota-driven pathogen refractoriness. *Staphylococcus*-based vaccines altered the microbiota composition but had no effect on *B. afzelii* infection, and yielded inconclusive results for *R. helvetica*. In contrast, previous studies suggest that *E. coli*-based microbiota modulation can induce a pathogen-refractory state, highlighting the importance of both bacterial species and peptide selection in shaping microbiota-driven pathogen susceptibility. However, a direct comparison under identical experimental conditions across multiple taxa is required to confirm this taxon-specific effect. These findings suggest that antimicrobiota vaccination influences tick fitness and microbiota assembly, but its effects on pathogen transmission depend on the bacterial taxon targeted and the selected peptide epitopes. This research provides insights into the need for strategic bacterial taxon selection to enhance vaccine efficacy in controlling tick-borne diseases.

## 1. Introduction

Tick-borne diseases (TBDs) pose significant public health challenges globally, with spirochetes from the *Borrelia burgdorferi* sensu lato complex, the causative agents of Lyme disease [1], being among the primary concerns [2,3]. While the pathogenicity of *Rickettsia helvetica* remains to be fully established [4], this bacterium serves as a valuable model for comparative studies with other highly virulent *Rickettsia* species within the spotted fever group, to which it belongs [5], as well as for other tick-borne pathogens (TBPs). The effective control of TBPs relies on understanding the complex interactions between the host, the pathogen, the vector, and the microbiota [6]. Indeed, recent research has focused on determining the role of the tick microbiota on pathogen acquisition and vector competence [7].

Vaccination strategies against arthropod-borne diseases have traditionally targeted the etiologic agent [8]. However, emerging evidence suggests that modifying the microbiota of vectors may offer an alternative or complementary approach to disease control [9]. Antimicrobiota vaccines, which target specific components of the vector microbial community [10], have the potential of disrupting infection [9], which may reduce the transmission rates. Despite this potential, the mechanisms through which antimicrobiota vaccines exert their effects [6,7], as well as their impacts on vector biology and microbiota, remain underexplored.

*Ixodes ricinus* is the most abundant tick in Europe and a vector of several TBPs, including bacteria in the complex *B. burgdorferi* sensu lato and *R. helvetica* [11,12]. Intriguingly, the presence of bacteria in the genus *Staphylococcus* was associated with the absence of pathogenic agents in *I. ricinus* [13]. As the tick microbiota influences vector competence [6], we hypothesized that *Staphylococcus* may exert a protective effect against infection through mechanisms such as competitive exclusion, the production of antimicrobial peptides, and/or the modulation of host immune responses, creating an unfavorable environment for the colonization of pathogenic agents. Therefore, in this study, we assessed the effects of immunization of C3H/HeN mice with either live *Staphylococcus epidermidis* bacteria or multi-antigenic peptides (MAPs). In order to obtain a peptide-based vaccine against *Staphylococcus* genus, the iron surface determinant B (IsdB) was chosen. IsdB is a known surface protein of *Staphyloccocus aureus* that plays the role of iron sequestering in an iron-limited condition [14]. Additionally, this protein is being used for the development of a multi-subunit vaccine against *S. aureus* [15], exerting a protective efficacy in animal models [16,17].

The effect on the tick fitness, pathogen acquisition, and microbiota composition and assembly was also assessed. Altogether, our results highlight the potential of microbiota-targeted interventions as a novel avenue for vector management, which may reduce the burden of TBDs.

## 2. Materials and Methods

### 2.1. Ethics Statement

All of the procedures were performed at the Animal Facility of the Laboratory for Animal Health of the French Agency for Food, Environmental and Occupational Health & Safety (ANSES), Maisons-Alfort, France, according to French and International Guiding Principles for Biomedical Research Involving Animals (2012). The procedures were reviewed and approved by the Ethics Committee (ComEth, Anses/ENVA/UPEC), with permit number E 94 046 08 for experiments with *Borrelia* and number 2023063016568996 for experiments with *Rickettsia*.

### 2.2. Mice and Housing Conditions

Six-week-old mice strain C3H/HeN (Charles River strain code 025) were purchased from Charles River (Miserey, France) and used to test the effects of antimicrobiota vaccines on either *Borrelia* (25 females) or *Rickettsia* (27 males) infection. Mice were kept for one week to acclimate before the experiment. All of the animals were maintained in green line ventilated racks (Tecniplast, Hohenpeissenberg, Germany) at −20 Pa, with food (Kliba nafaj, Rinaustrasse, Switzerland) and water ad libitum during the time of the experiment. The number of mice per cage was limited to five females or three males. Mice were kept at a controlled temperature (RT, 20–23 °C) and a 12 h (h) light/12-h dark photoperiod regimen. Animals were monitored twice a day (d) by experienced technicians, and deviations from normal behaviors or signs of health deterioration were recorded and reported.

### 2.3. Bacterial Cultures

Low-passage *Borrelia afzelii* CB43 were started from glycerol stocks and grown in Barbour–Stoenner–Kelly (BSK)-H (Sigma-Aldrich, St. Louis, MO, USA) media containing 6% rabbit serum, and were kept at 33 °C for 7 days, as detailed by [18]. *Rickettsia helvetica* was grown in Vero cells with MEM media (5% FBS; 1% Glutamine) following the procedures described by [19]. *Staphylococcus epidermidis* (ATCC-14990, LGC Limited, Teddington, Middlesex, UK) was grown in Tryptose Soy Broth (TSB; Sigma-Aldrich, St. Louis, MO, USA) at 37 °C under constant agitation at 100 rpm overnight.

### 2.4. Multi-Antigenic Peptide Design and Synthesis

In order to develop a peptide-based vaccine targeting the Staphylococcus genus, the iron surface determinant B (IsdB) was chosen as the foundation for the multi-antigenic peptide (MAP). IsdB is a surface protein of *Staphylococcus* involved in iron sequestration under iron-limited conditions [14]. This protein has been utilized in the development of a multi-subunit vaccine against *Staphylococcus* aureus, demonstrating protective efficacy in animal models [15,16,17]. For peptide selection, multiple criteria were analyzed. Firstly, B-cell epitope prediction was conducted using the ABCPred server (https://webs.iiitd.edu.in/raghava/abcpred/ (accessed on 1 February 2024)) [20,21], applying a threshold of 0.51 by default and a window length of 16. The antigenicity of potential epitopes was analyzed with VaxiJen v. 2.0 [22,23,24]. As the prediction threshold influences the accuracy, sensitivity, and specificity, a stringent threshold of 0.4 was applied. The allergenicity was predicted with AllerCatPro 2.0 [25]. Besides predicting the allergenicity, AllerCatPro also shows the potential cross-reactivity of the epitopes. The potential toxicity of the putative epitopes was evaluated with Toxinpred (https://webs.iiitd.edu.in/raghava/toxinpred/ (accessed on 1 February 2024)), and surface accessibility was evaluated with Emini Surface Accessibility prediction [26]. Finally, the coverage of multiple *Staphylococcus* species was conducted by blasting the candidate epitope using BLASTP [27] with the non-redundant protein sequences database; the filters excluded *S. aureus* (taxid:1280). The epitope with the best match in all of the applied parameters (highly antigenic, non-allergenic, and non-toxic, and covering multiple species) was used for the vaccine formulation. The physicochemical properties, such as the molecular weight, the isoelectric point, the half-life, the instability index, the aliphatic index, and the hydropathicity of the selected peptide, were conducted using the ProtParam tool [28].

The peptide containing the amino acid sequence IQDKLPEKLKAEYK was selected for its score of 0.64 for B-cell prediction, an antigenicity score of 0.87, and having no evidence of allergenicity, cross-reactivity, and toxicity. The sequence was aligned and found conserved in *S. epidermidis*, *S. argentus*, *S. schleiferi*, *S. aureus*, *Staphylococcus* sp. EG-SA-13, *Staphylococcus* sp. KY49P, *S. singaporensis*, *S. roterodami*, *Staphylococcus* sp. HMSC062H10, and *S. schweitzeri*. The selected epitope was used to synthetize a MAP. Each branch of the MAP had the selected *S. epidermidis* epitope (IQDKLPEKLKAEYK) fused to a universal T-helper cell epitope: the Pan DR epitope peptide (AKFVAAWTLKAAA) [29,30]. The individual branches were chemically ligated to the lysine core. The molecular mass of the synthetized MAPs was checked by Matrix-Assisted Laser Desorption Ionization—Time-of-Flight (MALDI/TOF) mass spectrometry. The MAP was synthetized with >80% purity (LifeTein, Somerset, NJ, USA). The vaccine was prepared using lyophilized MAPs rehydrated in sterile water.

### 2.5. Mouse Immunization

C3H/HeN mice were immunized subcutaneously with 100 µL of MAPs (10 µg per mouse) in a water-in-oil emulsion containing 75% Montanide™ ISA 71 VG adjuvant (Seppic, Paris, France), with two booster doses every 2 weeks after the first dose.

A live bacteria vaccine was prepared as previously described [31]. Briefly, *S. epidermidis* culture was washed with PBS (Thermo Scientific, Waltham, MA, USA), resuspended in sterile PBS, and homogenized using a glass homogenizer. Each mouse was immunized subcutaneously with 100 µL of *S. epidermidis* (1 × 10^7^ CFU) in a water-in-oil emulsion containing 75% Montanide™ ISA 71 VG adjuvant, with one booster dose 2 weeks after the first dose.

For both vaccine types, the control mice received a mock vaccine containing sterile PBS in the adjuvant.

### 2.6. Mouse Infection

For infection with *B. afzelii* CB43, 1 × 10^6^ spirochetes suspended in 250 µL of BSK-H media were inoculated subcutaneously (100 µL) and intraperitoneally (150 µL) into each mouse. Control mice were injected with BSK-H media alone, following the same protocol as described before [31].

For rickettsial infection, one vial of the inoculum (*R. helvetica*-infected Vero cells) in sterile PBS was heat-shocked with three cycles at 37 °C, followed by 30 s in liquid nitrogen (N_2_) to disrupt the Vero cells and release the rickettsiae. Then, 50 µL of the resulting cell suspension containing 4 × 10^9^ rickettsiae were inoculated intravenously through the retro-orbital sinus of each mouse. Control mice were injected with the same volume of heat-shocked pathogen-free Vero cells, following the same protocol as described in [19].

### 2.7. Tick Infestation

Immunized mice that were exposed to *Borrelia* were infested with pathogen-free unfed *I. ricinus* nymphs obtained from the colonies of UMR BIPAR (Maisons-Alfort, France), while the mice exposed to *Rickettsia* were infested with pathogen-free unfed *I. ricinus* nymphs obtained from Insect services (Berlin, Germany). The mice were anesthetized with isoflurane and a 2-cm-outer-diameter EVA foam capsule (Cosplay Shop, Bruges, Belgium) was fixed to their shaved backs using the non-irritating latex glue Tear Mender (LHB Industries, St. Louis, MO, USA), as described in [30]. Each mouse was infested with 25 *I. ricinus* nymphs at either d30 (after 2 vaccine shots for groups vaccinated with live *Staphylococcus*) or d42 (after 3 vaccine shots for groups vaccinated with MAPs). Tick feeding capsules were monitored twice a day. Engorged nymphs were collected, weighed, and stored in sterile tubes at −80 °C until use.

### 2.8. Sera Preparation

Approximately 100 μL of blood was collected from the mice of all experimental groups using a Pasteur pipette, via the retro-orbital sinus, without anticoagulant at d0, and every two weeks after vaccination until d30 for the live vaccine and d42 for the MAP vaccine. Blood samples were incubated for 2 h at room temperature (RT), allowing for clotting. The samples were centrifuged at RT for 5 min at 5000 rpm, and the resulting supernatant was centrifuged again with the same conditions. The resulting supernatant (sera) was transferred to a clean sterile tube and stored at −20 °C until use.

### 2.9. Bacterial Protein Extraction

An amount of 7 mL of a *B. afzelii* suspension, with a density of at least 1 × 10^7^ per mL, was centrifuged at 8000 rpm for 10 min at 20 °C. The resulting pellet was washed twice with 1 mL of cold HN-Buffer and centrifuged at 8000 rpm for 10 min at 20 °C. The resulting pellet was resuspended in 200 µL of bacterial protein extraction buffer (B-PER) (Thermo Scientific, Waltham, MA, USA), incubated at RT for 10 min, and stored at −20 °C until use.

The *Staphylococcus epidermidis* suspension was centrifuged at 4 °C for 5 min at 1000× *g*. The resulting pellet was washed twice with 1 mL of sterile PBS. The bacterial pellet was resuspended in PBS containing 1% Triton (Sigma-Aldrich, St. Louis, MO, USA). The sample was centrifuged for 5 min at 200× *g* to remove the debris, and the resulting supernatant was conserved at −20 °C until use.

### 2.10. Western Blot

Western blotting was performed to evaluate the presence of anti-*Borrelia* antibodies in the mouse sera. The protein extract of *B. afzelii* was mixed with an equal volume of 2× Laemmli Buffer (Thermo Scientific, Waltham, MA, USA) and heated at 100 °C for 10 min for protein denaturation. An amount of 20 µg of protein was loaded in each lane of a 4–15% Mini-PROTEAN TGX Stain-Free Protein gel (Bio-Rad, Hercules, CA, USA). Electrophoresis was run at 120 V for 1 h. The proteins were then transferred onto a nitrocellulose membrane (Bio-Rad, Hercules, CA, USA) using a semidry transfer method. Blotting was performed for 30 min at 25 V in a transfer cell (Trans-Blot SD, Bio-Rad, Hercules, CA, USA). The membrane was incubated with 1% bovine serum albumin (BSA)/PBS (Sigma-Aldrich, St. Louis, MO, USA) for 2 h at RT. After the blocking step, the membrane was incubated overnight at 4 °C with mouse sera at a dilution of 1:100 in PBS. Then, the membrane was washed three times with PBS tween (0.5%) for 10 min each with gentle agitation. After incubation with HRP-conjugated antibodies (Abs, goat anti-mouse IgG) (Sigma-Aldrich, St. Louis, MO, USA) diluted at 1:2000 in PBS for 1 h at RT, the membrane was washed three times with the same conditions described above. The antibody detection was performed by chemiluminescence using a Pierce ECL Western blotting substrate (BioRad, Hercules, CA, USA). After an incubation for 3 min, the membrane was analyzed using a ChemiDoc™ Touch Imaging System (Bio-Rad, Hercules, CA, USA).

### 2.11. Indirect ELISA

The levels of Abs that were reactive against bacterial proteins were measured in the mice sera, as previously reported [31]. An amount of 50 ng of the protein extract of *S. epidermidis* or of the MAPs in 100 µL of coating carbonate/bicarbonate buffer (0.05 M, pH 9.5) were loaded to each well of a 96-well ELISA plate (Thermo Scientifc, Waltham, MA, USA) overnight at 4 °C. For the first incubation, 50 µL of sera from either the control mice or mice immunized with either live *S. epidermidis* or MAPs (diluted 1:700 in PBS) were loaded to each well and incubated for 1 h at 37 °C under gentle agitation. The secondary antibody (anti-mouse IgG or IgM), diluted at 1:1500 in sterile PBS, was loaded to each well for 1 h at RT under gentle agitation. An amount of 100 µL of a ready-to-use TMB solution (Promega, Madison, WI, USA) was applied per well and, after 20 min at RT under gentle agitation, the reaction was stopped by the addition of 50 µL of 0.5 M sulfuric acid. The absorbance at 450 nm was determined using a spectrophotometer (Filter-Max F5, Molecular Devices, San Jose, CA, USA). The absorbance of the blank (without the sera addition) was subtracted from the absorbance of the samples. The serum of each mouse was analyzed in technical triplicates.

### 2.12. Complement and Serum Bactericidal Activity Assay

A baby rabbit complement (BRC, Bio-Rad) solution (final concentration of 2.5%; *v*:*v*) was mixed with an *S. epidermidis* suspension, diluted 200,000 times. Sera samples of each mouse group (Mock, Live, or MAP vaccine groups) were pooled and incubated at 56 °C for 30 min. For each group, the heat-inactivated sera were mixed to the suspension of *S. epidermidis* and BRC (with a final concentration of 5%). A control group containing only *S. epidermidis* and BRC was used as the growth reference. The bacterial suspensions were incubated at 37 °C and aliquots collected after 1, 2, 3, and 4 h were spread on Tryptic Soy Agar (Sigma-Aldrich, St. Louis, MO, USA). After an overnight incubation of the plates at 37 °C, the number of colony-forming units (CFUs) was recorded.

### 2.13. DNA Extraction

Genomic DNA (gDNA) was extracted from engorged nymphs and from mice spleens using the Nucleospin tissue DNA extraction Kit (Macherey-Nagel, Hoerdt, France), and then eluted in either 30 µL (tick samples) or 50 µL of sterile water, respectively. The gDNA concentration was determined using a NanoDrop™ One (Thermo Scientific, Waltham, MA, USA). As a control, five samples without any biological material were processed as described above.

### 2.14. Quantification of B. afzelii Load by qPCR

For the detection of *B. afzelii* in the tick tissues, a pre-amplification step and a quantitative PCR (qPCR) were performed, as described in [32]. For the pre-amplification, the primers targeting the 23S rRNA gene of *Borrelia* spp. (23S rRNA-F: GAGTCTTAAAAGGGCGATTTAGT; 23S rRNA-R: CTTCAGCCTGGCCATAAATAG) were used. For the qPCR, each reaction contained 6 μL of FastStart universal probe master (Roche, Meylan, France), 0.12 μL of 20 µM of the same aforementioned primers and a TaqMan 23S rRNA-probe (23S rRNA-probe, ‘AGATGTGGTAGACCCGAAGCCGAGT’), 2 μL of the pre-amplified DNA, and Milli-Q ultrapure water up to 12 µL. The amplification program of 95 °C for 5 min, and 45 cycles of 95 °C for 10 s, and 60 °C for 15 s, was performed by a LightCycler 480 (Roche, Meylan, France). The spirochete load in each tick sample was obtained by the interpolation of the Cq value in a standard curve of the number of spirochetes vs. Cq, and then was normalized by the quantity of DNA in each sample.

### 2.15. Quantification of R. helvetica Load by Conventional PCR and qPCR

For the detection of *R. helvetica* in mice, a conventional PCR was performed using the gDNA extracted from mice spleens as a template. The primers for the citrate synthase-encoding gene (*gltA*; CS78: 5′ GAG AGA AAA TTA TAT CCA AAT GTT GAT 3′ and CS283 5′ AGG GTC TTC GTG CAT TTC TT 3′) were used. Reactions were performed in a final volume of 50 µL containing 5 µL of buffer 10×, 4 µL of dNTPs, 1 µL of a 10 μM solution of each primer, 0.25 µL of Taq polymerase (Takara; Thermo Scientific, Waltham, MA, USA), 36.75 µL of distilled water, and 2 µL of DNA template. The thermocycling program consisted of an initial step at 95 °C for 2 min, 40 cycles at 95 °C for 10 s, 48 °C for 30 s, and 72 °C for 10 s, and one extension incubation at 72 °C for 3 min. The amplicons were separated by electrophoresis on a 2% agarose gel, stained with ethidium bromide (final concentration of 0.5 μg/mL), and visualized using a transilluminator, as explained in [19].

For the detection of *R. helvetica* in ticks, 50 samples per condition were randomly selected for the qPCR analysis. The reaction contained 6 µL of Master mix (Roche, Meylan, France), 0.25 µL of a 10 μM solution of each primer, 0.5 µL of distilled water, and 5 µL of the DNA template. The program consisted of one step of 5 min at 95 °C followed by 45 cycles of 10 s at 95 °C, 15 s at 55 °C, and 15 s at 72 °C. The quantitation cycle (Cq) of each sample was determined by LightCycler 480 software (Roche, Meylan, France), compared with the Cq of a standard curve constructed with serial dilutions of the *gltA* amplicon, as detailed by Galletti et al. [32], and used to calculate the number of genomic equivalents of *Rickettsia* per µL of gDNA sample.

### 2.16. V4 Region 16S rRNA Amplicon Sequencing and Processing of Raw Sequences

The gDNA extracted from the nymphs (200 ng) was used for the next-generation sequencing, which was commissioned by Novogene Bioinformatics Technology Co. (London, UK). A single lane of the Illumina MiSeq system was used to generate 251-base paired-end reads of the V4 variable region of the bacterial 16S rRNA gene using bar-coded universal primers (515F/806R). DNA samples from nonexposed nymphs vaccinated with mock (Mock, *n* = 23), nonexposed nymphs vaccinated with MAPs (MAPs, *n* = 24), nonexposed nymphs vaccinated with live *Staphylococcus* (Live, *n* = 12), *B. afzelii*-exposed nymphs vaccinated with mock (Mock + *Borrelia*, *n* = 10), *B. afzelii*-exposed nymphs vaccinated with MAPs (MAPs + *Borrelia*, *n* = 14), *B. afzelii*-exposed nymphs vaccinated with live *Staphylococcus* (Live + *Borrelia*, *n* = 10), *R. helvetica*-exposed nymphs vaccinated with mock (Mock + *Rickettsia*, *n* = 12), *R. helvetica*-exposed nymphs vaccinated with MAPs (MAPs + *Rickettsia*, *n* = 14), *R. helvetica*-exposed nymphs vaccinated with live *Staphylococcus* (Live + *Rickettsia*, *n* = 9), and extraction reagent control (*n* = 5) were used. The sequences are accessible in NCBI bioproject No. PRJNA1145395. The 16S rRNA gene sequences were analyzed using the Quantitative Insights Into Microbial Ecology 2 (QIIME2) pipeline (v.2022.8) [33]. The demultiplexed raw sequences (obtained in fastq files) were denoised, quality-trimmed, and merged using DADA2 software (version 1.26.0) [34], implemented in QIIME 2 [33]. The reads were then merged, chimeric variants were removed, and then were taxonomically assigned using a pre-trained naïve Bayes taxonomic classifier [35] based on the SILVA database (v. 138) [36]. The resulting taxonomic table was collapsed at the genus level and filtered by removing taxa with less than 10 reads, or which were present in less than 30% of samples. Possible contaminating DNA in the samples for the 16S rRNA gene sequencing was statistically identified with the ‘decontam’ package [37] using the ‘prevalence’ method. The prevalence method is defined as the presence or absence across samples. A contaminant is a sequence that is more prevalent in the negative control than in the samples, with a threshold of 0.05. Then, the contaminants were removed from the dataset before the downstream microbiota analysis [37].

### 2.17. Microbial Diversity

The alpha diversity metric calculates the diversity within the sample by measuring the richness and evenness of the bacterial community. The richness was measured with observed features [38], and the evenness with Pielou’s evenness [39], both estimated using the q2-diversity plugin implemented on QIIME2 [33]. The beta diversity metric calculates the diversity of the microbiota between conditions. The beta diversity was observed with the Bray–Curtis dissimilarity test [40], estimated using the q2-diversity plugin implemented on QIIME2 [33]. The beta dispersion was tested for the three conditions using the ‘betadisper’ function in the Vegan package [41].

The microbial composition of the three vaccinated groups was analyzed with an upset graph. This graph represents shared and unique taxa in each group, as well as the size of the microbial community for each condition. The upset graph was built with the “UpSetR” package [42] in R (v. 4.1.2) [43]. The abundance of each microbial taxa was compared between the three conditions. Firstly, the data were transformed into centered log ratio (clr) values, utilizing the geometric mean of the read counts in each sample to assess relative abundance [44]. The clr value transformation was performed with the ‘ANOVA-like differential expression’ (‘ALDEx2′) package [45] in the R program (v.4.1.2) [43].

### 2.18. Bacterial Co-Occurrence Network Construction

For each condition, microbial co-occurrence networks were constructed using the taxonomic table. Networks were built using the ‘Sparse Correlations for Compositional data’ (‘SparCC’) method [46], implemented in the R program (v.4.1.2) [43]. Edge significances were tested with the bootstrap method (B = 1000), and only edges with a corrected *p*-value < 0.05 and with a cutoff of >0.75 for positive correlations and <−0.75 for negative ones were considered for the co-occurrence networks. Networks were visualized and topological features (i.e., the number of nodes and edges, modularity, network diameter average, and weighted degree and clustering coefficient) were calculated with Gephi 0.9.2 [47].

### 2.19. Network Robustness

Robustness evaluation was conducted using node addition and removal. The loss of connectivity induced by the removal of a fraction of nodes was calculated using the NetSwan [48] script, implemented in the R program (v.4.1.2) [43]. Nodes with high betweenness centrality (recalculated at each removal) were removed first.

The largest connected component (LCC) size and the average path length were (APL) were calculated for each condition, with the addition of 10 to 100 nodes (with a step of 10 nodes), using the method from Freitas et al. [49], implemented in the R program (v.4.1.2) [43]. The results were visualized using GraphPad Prism (v. 9.0.2) (GraphPad Software, San Diego, CA, USA).

### 2.20. Effect of the Vaccines on Microbial Assembly of Pathogen-Exposed Ticks

The microbiota of *I. ricinus* ticks exposed to either *Borrelia* or *Rickettsia* was compared for the vaccinated groups. Co-occurrence networks were built with the same method described above (SPARCC weight > 0.75 or <−0.75) and visualized with Gephi 0.9.2 [47].

### 2.21. Comparison of Infection-Refractory and Infection-Permissive States of the Microbiota

We compared the microbial states of the ticks considered as ‘infection-refractory’ or ‘infection-permissive’ states to infection with *Borrelia* and *Rickettsia.* For *Borrelia*, we sourced data on the ‘infection-refractory’ state from Bioproject No. PRJNA1065249, where the researchers investigated the impact of *Escherichia coli* and its antibodies on the *B. afzelii* levels and the microbiota of *I. ricinus* ticks [18]. These data were compared with the data on the ‘infection-permissive’ state of the microbiota obtained by the current study. The sequences were collected from the SRA repository and analyzed with the same pipeline as explained before. The state of the microbiota, described as the intersection between the bacterial diversity and microbial interactions, were compared with the number of observed features in the function of the number of nodes and edges. This information was obtained with QIIME 2 [33] and Gephi 0.9.2 [47].

For *Rickettsia*, the data on the ‘infection-refractory’ state of the microbiota were obtained from the current study. Data on the ‘infection-permissive’ state of the microbiota were obtained from another study [13], in which the microbiota of *I. ricinus* ticks naturally infected with *R. helvetica* collected from humans was assessed. The sequences were collected from the Bioproject No. PRJNA803003, and the sequences were analyzed with the same pipeline as explained before. The state of the microbiota was compared with the number of observed features in the function of the number of nodes and edges. This information was obtained with QIIME 2 [33] and Gephi 0.9.2 [47].

### 2.22. Statistical Tests

The comparison of the anti-*S. epidermidis* and anti-MAP IgG and IgM levels was performed at the final time point with Šídák’s multiple comparison test (*p* < 0.05) conducted in Graphpad Prism v. 9.0.2. The comparison of the number of *S. epidermidis* CFUs at the final time point (4 h) was conducted with Tukey’s multiple comparison test (*p* < 0.05) with Prism v. 9.0.2. The tick fitness comparison was assessed with an unpaired *t*-test (*p* < 0.05) for the weight, a log-rank (Mantel–Cox) test (*p* < 0.05) for the feeding rate, and an unpaired *t*-test (*p* < 0.05) for the survival rate with Prism v. 9.0.2. The comparison of the number of infected ticks with *B. afzelii* or *R. helvetica* was performed with a Chi-squared test (*p* < 0.05), and the comparison of the number of spirochetes per ng of DNA was performed with an unpaired *t*-test (*p* < 0.05) in Prism v. 9.0.2. The alpha diversity of the microbiota of the MAP, Live, and Mock vaccine groups were statistically compared with a pairwise Kruskal–Wallis test (*p* < 0.05). The beta diversity of the microbiota of the MAP, Live, and Mock vaccine groups were statistically compared with a pairwise permanova test (*p* < 0.05). An ANOVA (*p* < 0.05) test was performed to statistically compare the dispersion of the microbial samples of the three conditions. For the comparison of the taxa abundance, the clr values were statistically compared using a Kruskal–Wallis test (*p* < 0.05) with the Benjamini–Hochberg correction [50]. The statistical analysis was performed with the ‘ALDEx2’ package [45] in the R program (v.4.1.2) [43]. Significantly different taxa were highlighted in a heatmap produced in GraphPad Prism (v. 9.0.2) (GraphPad Software, San Diego, CA, USA).

## 3. Results

### 3.1. Mouse Response to Staphylococcus-Based Vaccines

**Figure 1:**
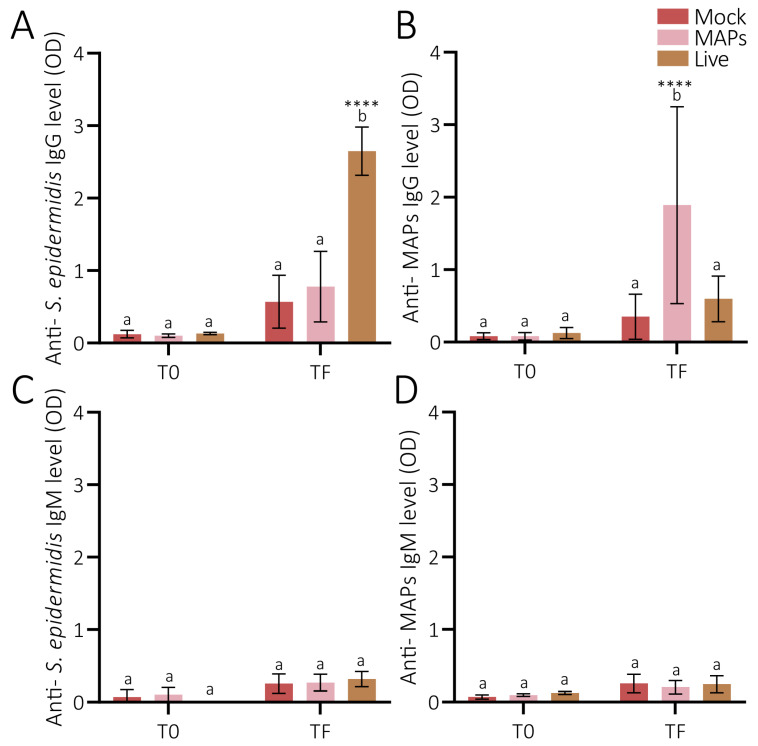
**Effects of the different vaccine formulations on the production of antibodies by mice**. Mean levels of IgGs and IgM anti-*Staphylococcus epidermidis* proteins ((**A**) and (**C**), respectively) and anti-MAP *Staphylococcus* ((**B**) and (**D**), respectively) in the sera of mice vaccinated with Mock (red), MAPs (pink), or Live *S. epidermidis* (brown). Error bars represent the standard deviations. Šídák’s multiple comparisons test was performed to analyze the antibody statistical differences between time 0 (T0, right before the first immunization) and time final (TF, 30 days for the Live group and 42 days for the MAP group after the first immunization) for each group of vaccinated mice, and among the vaccinated mice of each time point. The same letters mean non-significant tests; different letters mean significant differences (****: *p* < 0.0001).

The immunization of mice with live *S. epidermidis* stimulated the production of antibodies against the protein extracted from *S. epidermidis*, with significantly higher levels of IgGs than the mock group (Šídák’s multiple comparisons, *p* (time final (TF, 30 days for live and 42 days for the MAP group after the first immunization), Live vs. Mock) <0.0001; Figure 1A). Similarly, the levels of *Staphylococcus* MAP IgGs were significantly higher in the mice immunized with MAPs than in those of the Mock group (Šídák’s multiple comparisons, *p* (TF MAPs vs. Mock) = 0.0015; Figure 1B). Moreover, a high variability was observed in the levels of *Staphylococcus* MAP IgGs for the MAP group (Figure 1B). No cross-reactivity of the IgG against the *S. epidermidis* protein for the MAP-vaccinated mice (Šídák’s multiple comparisons, *p* (TF, MAPs vs. Mock) = 0.5050; Figure 1A) or of anti-MAP IgGs for live-vaccinated mice (Šídák’s multiple comparisons, *p* (TF Live vs. Mock) = 0.7924; Figure 1B) was observed. In addition, no significant differences were observed in the levels of IgM in mice immunized with either live *S. epidermidis* (Figure 1C) or MAPs (Figure 1D) in relation to the control.

**Figure 2:**
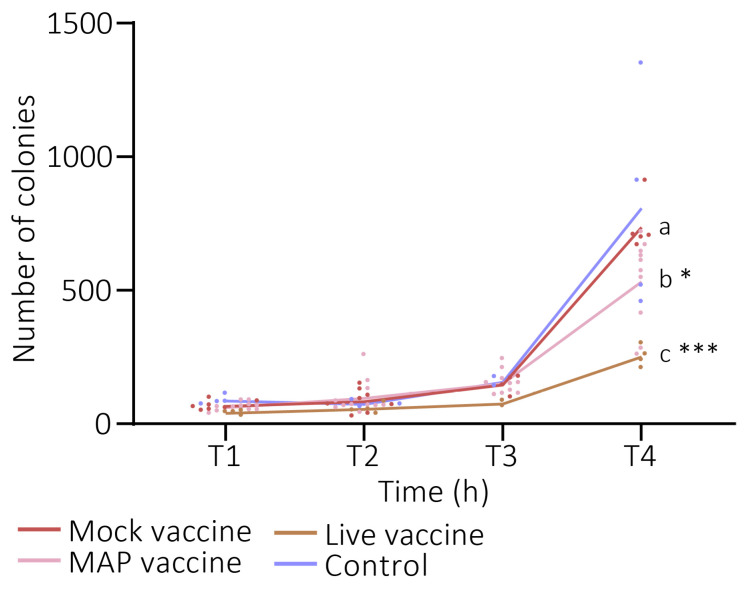
**Serum bactericidal effect on *S. epidermidis*.** The number of *S. epidermidis* CFUs after 1, 2, 3, and 4 h in contact with the Mock-, MAP-, and Live-vaccinated mice sera, or in the absence of sera (control). Each dot represents a replicate and each line represent the mean value. Tukey’s multiple comparisons test was performed for the number of CFUs in the final time point. The same letters mean no significant differences, and different letters mean significant differences (*: *p* < 0.05; ***: *p* < 0.001).

The bactericidal effect of the complement-inactivated sera of mice of the Mock, MAP, and Live groups were compared. The number of colonies of *S. epidermidis* were measured after prolonged contact with the sera. The control group (the bacteria incubated only with the baby rabbit complement) and the Mock group had similar levels of colonies after 4 h of incubation. However, both the Live and MAP groups affected the number of colonies of *S. epidermidis* compared with the Mock group (Tukey’s multiple comparisons, *p* (T4, Live vs. Mock) = 0.0002; *p* (T4, MAPs vs. Mock) = 0.0256; Figure 2).

### 3.2. Effects of the Mouse Vaccination on Tick Fitness

**Figure 3:**
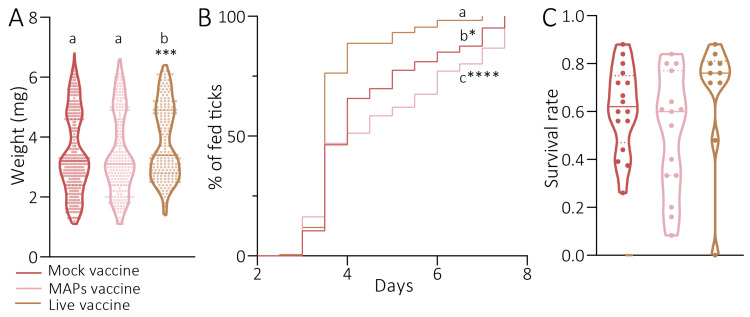
**Effects of microbial modulations on tick feeding fitness and survival rate.** (**A**) Weight (mg), (**B**) the percentage of engorged nymphs along feeding, and (**C**) the survival rate of *I. ricinus* nymphs fed on the control mice (brown) or mice immunized with either live *S. epidermidis* (pink) or MAPs (red). Each dot represents one specimen, the lines represent the median value and the dashed line represents the quartiles. Statistical tests were performed between the three groups to test significant differences in the weight (unpaired *t*-test), feeding rate (Mantel–Cox test), and survival rate (unpaired *t*-test). The same letters means that there is no significant difference (*p* > 0.05), while different letters mean that a significant difference was observed between values (* for *p* < 0.05; *** for *p* < 0.001; **** for *p* < 0.0001).

The nymphs fed on mice immunized with live *S. epidermidis* showed a significantly higher weights compared with the two other groups (unpaired *t*-test, Mock vs. Live, *p* = 0.0008; MAPs vs. Live, *p* = 0.0009; Figure 3A). No significant differences were observed between the weights of the nymphs fed on mice from the Mock and MAP groups (unpaired *t*-test, Mock vs. MAPs, *p* = 0.8388; Figure 3A). The nymphs also exhibited three different feeding patterns, depending on the group of mice they fed on (Figure 3B). The nymphs fed on mice immunized with live vaccine had a significantly quicker feeding time compared with the Mock group. While most of the nymphs in the vaccine group were engorged by d4, less than 75% of the nymphs of the Mock group had fed at the same point (Mantel–Cox test, Mock vs. Live, *p* < 0.0001; Figure 3B). Engorgement differences were also observed between the nymphs fed on mice immunized with live vaccine in relation to those fed on mice immunized with the MAP vaccine, where less than 50% of the nymphs fed by d4 (MAPs vs. Live, *p* < 0.0001; Mock vs. MAPs, *p* = 0.0131; Figure 3B). In relation to the survival of the nymphs, those fed on mice immunized with MAPs and Mock exhibited the lowest survival rate among all of the groups, only 60%, compared with those fed on mice immunized with live vaccine (80%); however, the differences were not significant (unpaired *t*-test, *p* > 0.05; Figure 3C).

### 3.3. Detection of Pathogens on Mice

The Western blot analysis conducted on the sera of mice infected with *B. afzelii* demonstrated a distinct mark on all of the samples infected by *Borrelia* (Supplementary Figure S1). The infection of *R. helvetica* was confirmed by conventional PCR in all of the mice (6/6) from the mock-vaccinated group, in 67% (4/6) of the mice vaccinated with the live vaccine, and in 83% (5/6) of the mice vaccinated with the MAP vaccine (Supplementary Figure S2). For the subsequent analyses, only ticks collected on mice that were positive by conventional PCR for the groups infected with *R. helvetica* were analyzed.

### 3.4. Effects of the Mouse Vaccination on the Acquisition of Pathogens by Ticks

**Figure 4:**
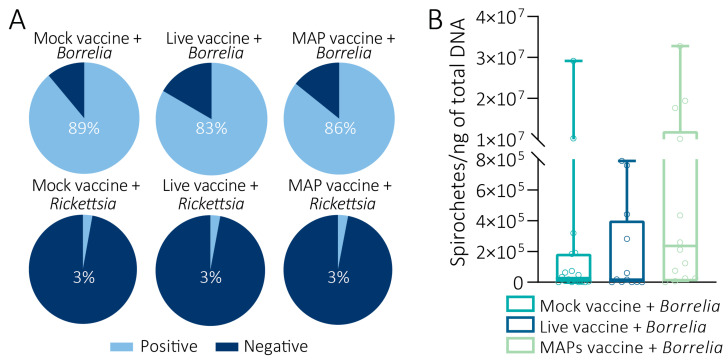
**Effect of vaccination on pathogen acquisition by ticks.** (**A**) Percentage of nymphs positive for *Borrelia* (on the top) or *Rickettsia* (on the bottom). The light blue in the charts represents the proportion of ticks positive for infection and dark blue the negative ones. (**B**) Number of spirochetes per ng of DNA in *I. ricinus* nymphs. No significant differences were observed in the proportion of infected ticks and the number of *Borrelia* spirochete per ng of total DNA between the groups (unpaired *t*-test).

The prevalence of ticks infected with *Borrelia* was not significantly different among the experimental groups, with 83% of infected nymphs when they fed on mice immunized with live vaccine, 86% in those fed on mice immunized with MAPs, and 89% in those fed on mice of the control group (Chi-squared test, *p* = 0.9066; Figure 4A). In relation to the load of spirochetes, no significant differences were observed among the different groups (unpaired *t*-test, *p* > 0.05; Figure 4B). The median borrelial level was the highest in the ticks immunized with MAPs (2.6 × 10^5^ for MAPs, 3.4 × 10^4^ for Mock, and 1.8 × 10^4^ for Live; Figure 4B). In addition, the ticks that fed on mice immunized with the live vaccine did not exhibit borrelial levels above 8 × 10^5^, as observed in the ticks that fed on the mice of the other groups (two nymphs above 1 × 10^7^ in the Mock group and four nymphs above 1 × 10^7^ in the MAP group; Figure 4B). A prevalence of only 3% of nymphs infected with *Rickettsia* was observed in the different groups (Chi-squared test, *p* = 0.9991; Figure 4A). Importantly, none of the ticks that fed on non-infected mice tested positive for *Borrelia* or *Rickettsia*.

### 3.5. Effect of the Vaccines on the Tick Microbiota

**Figure 5:**
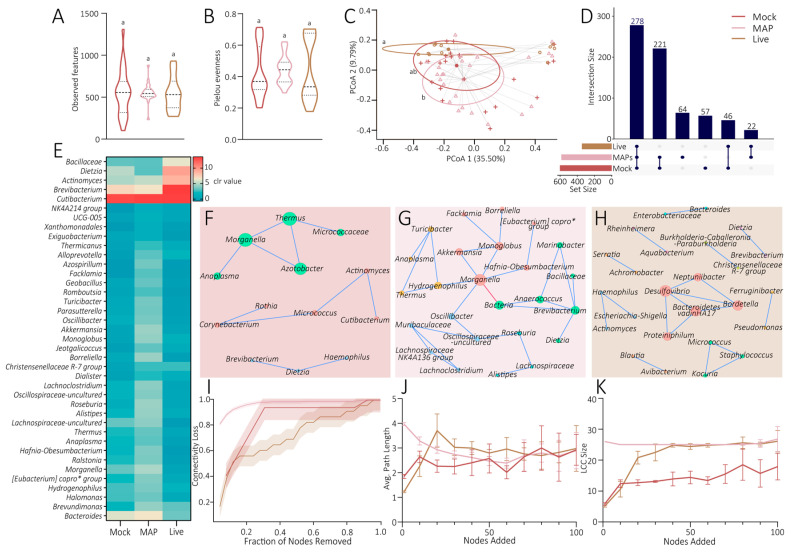
**Microbiota comparison of *I. ricinus* nymphs fed on mice immunized with the Mock, MAP, and Live vaccines.** Comparison of the alpha diversity with (**A**) observed features and (**B**) Pielou evenness. The dashed line represents the average value, and each dotted line represents the quartiles. Comparison of the beta diversity with (**C**) the Bray–Curtis dissimilarity index represented in a Principal Coordinate Analysis graph. The dots represent the microbial samples, the circles represent the dispersion of the samples, and the full dots represent the centroids of the dispersion circles. Statistical tests were performed between the three vaccine conditions to test the significant differences in the observed features (pairwise Kruskal–Wallis test), the evenness (pairwise Kruskal–Wallis test), the Bray–Curtis dissimilarity (pairwise permanova test), and the beta dispersion (ANOVA test). The same letters mean that there is no significant difference (*p* > 0.05), while different letters mean that a significant difference was observed between the values (*p* < 0.05). (**D**) Upset graph presenting the common and unique taxa of Mock, Live, and MAP groups. (**E**) A heatmap representing the taxa with significant different abundances (Kruskal–Wallis with the Benjamini–Hochberg correction test, *p* < 0.05) between the groups. Each line represents one taxon, and each column represents one condition. The clr value ranged from 0 (blue) to 13 (red). Copro* = coprostanoligenes. Microbial networks of (**F**) the Mock group, (**G**) the MAP group, and (**H**) the Live group. Each node represents a taxon, while each line represents a positive (weight > 0.75), in blue, and a negative (weight < −0.75), in red, co-occurrence interaction. The node size represents the eigenvector centrality value (a big node means a high eigenvector centrality value), and the node color represent a module (the same color means the same module). Copro* = coprostanoligenes. The robustness of the microbial networks tested with (**I**) the connectivity loss, depending of the fraction of the node removed with the highest betweenness centrality taxa first, (**J**) the average path length (Avg. Path Length), and (**K**) the largest connected component (LCC) size, depending on the nodes added. The lines represent the corresponding value; colored bands or brackets represent the confidence interval.

To assess the effect of vaccination on the vector microbiota, the alpha and beta diversities of the bacterial components of ticks fed on mice immunized with live *S. epidermidis*, MAPs, or the control (Mock) were calculated (Figure 5). The observed features did not demonstrate a significant difference in the richness of the different groups (pairwise Kruskal–Wallis test, *p* > 0.05; Figure 5A), as well as the Pielou’s evenness test (pairwise Kruskal–Wallis test, *p* > 0.05; Figure 5B). These results indicate that there is no substantial effect of the vaccination on the alpha diversity of the tick microbiota. For the beta diversity, no significant differences were observed for the Bray–Curtis dissimilarity test for the Mock vaccine group in relation to the two other groups (pairwise permanova, *p* > 0.05; Figure 5C). However, a significant difference was found between the MAP and Live vaccine groups (pairwise permanova, *p* = 0.010; Figure 5C). No significant difference was found on the dispersion of samples among the conditions (ANOVA, *p* > 0.05; Figure 5C). The microbiota of the ticks fed on mice immunized with live *S. epidermidis* exhibited a lower diversity of microbial taxa (346 taxa) compared with those fed on MAP-immunized (602 taxa) or Mock-immunized (586 taxa) mice (Figure 5D and Supplementary Table S1). A total of 278 microbial taxa are common between the Mock, Live, and MAP groups, and 221 taxa are shared only by ticks fed on mice immunized with the MAP and Mock vaccines. In addition, 64 and 57 taxa are unique to ticks fed on mice immunized with MAPs and the control, respectively. Ticks fed on mice immunized with live bacteria shared 46 taxa with the control, and 22 with the MAP group (Figure 5D; Supplementary Table S1). Regarding the abundance, 39 taxa exhibited significant differences among the three groups (Kruskal–Wallis with the Benjamini–Hochberg correction, *p* < 0.05; Figure 5E). Among them, six were more abundant in the ticks fed on live bacteria compared with the two other groups (Bacillaceae, *Dietzia*, *Actinomyces*, *Brevibacterium*, *Cutibacterium*, and Christensenellaceae *R-7* group) and 33 were more abundant in those fed on mice from the MAP group compared with the two other groups (*NK4A214* group, *UCG-005*, Xanthomonadales, *Exiguobacterium*, *Thermicanus*, *Alloprevotella*, *Azospirillum*, *Facklamia*, *Geobacillus*, *Romboutsia*, *Turicibacter*, *Parasutterella*, *Oscillibacter*, *Akkermansia*, *Monoglobus*, *Jeotgalicoccus*, *Borreliella*, *Dialister*, *Lachnoclostridium*, Oscillospiraceae uncultured, *Roseburia*, *Alistipes*, Lachnospiraceae-uncultured, *Thermus*, *Anaplasma*, *Hafnia-Obesumbacterium*, *Ralstonia*, *Morganella*, [Eubacterium] *coprostanoligenes* group, *Hydrogenophilus*, *Halomonas*, *Brevundimonas*, and *Bacteroides*; Figure 5E). The *Staphylococcus* taxa did not demonstrate a different relative abundance between the conditions.

The microbial networks of ticks from the three groups present dissimilarities in their structure (Figure 5F–H). The ticks of the Mock group present three communities not connected to each other, the lowest number of nodes and edges, and a low clustering coefficient, indicating a relatively small but loosely connected network with fewer community structures (Figure 5F; Table 1).

The network of ticks of the MAP group had its members fully connected and separated into five communities (Figure 5G; Table 1). The taxon *Morganella* was positioned in the center of the microbial network, having an important role in the network stability (Figure 5G). Moreover, this network presented the highest average degree, average path length, diameter, and number of links (Table 1), suggesting a more complex and robust network. The network of ticks of the Live vaccine group presented the highest modularity, with ten communities, suggesting a more complex structure (Figure 5H; Table 1). It had the shortest average path length and diameter, indicating a compact but highly modular and clustered network (Table 1). The robustness tests revealed that the network of the MAP group was highly vulnerable to node removal by cascading, as it reached 80% of connectivity loss with the removal of its nodes, with the highest betweenness centrality (Figure 5I). Indeed, the removal of the *Morganella* taxon, which has the highest betweenness centrality score (betweenness centrality = 220), significantly disrupted the microbial structure. Conversely, the Live vaccine group network was the most resilient to node removal (Figure 5I). However, the network response to node addition was similar for the networks of the three groups in relation to the average path length (Figure 5J). The networks of the two immunized groups (Live *S. epidermidis* or MAPs) had a similar response to the node addition regarding the LCC size, reaching a plateau at 26, while the network of the Mock group had a lower LCC size (between 12 and 16), suggesting a higher vulnerability to perturbations (Figure 5K).

### 3.6. Effect of the Vaccines on the Microbial Assembly of Infected Ticks

**Figure 6:**
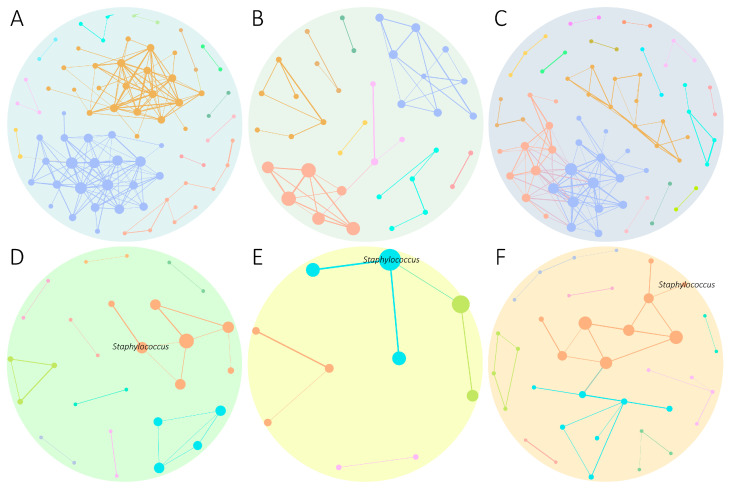
Microbial networks of *I. ricinus* ticks infected by *Borrelia* or *Rickettsia* and immunized with the Mock, MAP, and Live vaccines. Microbial network of (**A**) Mock, (**B**) MAP, and (**C**) Live groups infected with *B. afzelii*, and the (**D**) Mock, (**E**) MAP, and (**F**) Live groups exposed to *R. helvetica*. Nodes represent microbial taxa and edges represent positive interactions (SPARCC > 0.75). The node color and size represent the module and eigenvector centrality value, respectively. The edge thickness represents the weight (between 0.75 and 1).

The comparison of the bacterial assembly of infected groups of microbial networks demonstrated that no taxa from the Mock + *Borrelia* (Figure 6A), MAPs + *Borrelia* (Figure 6B), and Live + *Borrelia* (Figure 6C) harbored co-occurrence interactions with *Staphylococcus* or *Borrelia* taxa in their microbiota. A decrease in the number of edges was observed in the MAPs + *Borrelia* group, and a change in the modularity was observed in the Live + *Borrelia* group in comparison with the Mock + *Borrelia* group (Figure 6A–C).

*Staphylococcus* was a member of the networks of the microbiota of ticks fed on mice vaccinated with Mock, MAP, or Live vaccines and exposed to *Rickettsia*, constituting an important module (Figure 6D–F). In the Mock and MAPs + *Rickettsia* groups, *Staphylococcus* was in the center of its module (Figure 6D,E), while, in the Live + *Rickettsia* group, it was positioned on the periphery (Figure 6F).

### 3.7. Comparison of Infection-Refractory and Infection-Permissive States of the Microbiota

**Figure 7:**
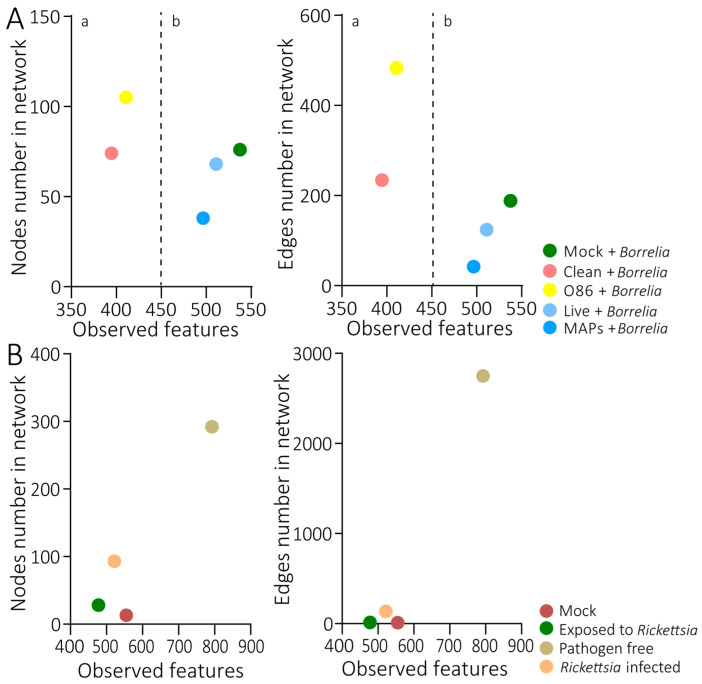
**Microbial structural states of ticks infected with *B. afzelii* or *R. helvetica* from different experimental conditions.** Scatter plot showing the mean of observed features versus the number of connected nodes and edges found in the microbial co-occurrence networks of ticks infected with (**A**) *Borrelia* or (**B**) *Rickettsia.* The different quadrants in the plot represent (a) the infection-refractory state and (b) the infection-permissive state.

To evaluate the correlation between the microbial modulations and pathogen loads, published microbial data with contrasting *Borrelia* and *Rickettsia* loads were used. The comparison of the states of the microbiota with the vaccination of *E. coli* or *S. epidermidis* revealed distinctive patterns within these states (a and b; Figure 7A). The Mock + *Borrelia*, MAPs + *Borrelia*, and Live + *Borrelia* groups displayed a decreased number of nodes and edges, despite having a similarly high number of observed features (Figure 7A). Notably, the Clean and *E. coli* O86:B7 groups demonstrated reduced numbers of observed features. Specifically, the Live + *Borrelia* and the MAPs + *Borrelia* groups are closely clustered with each other, and have been defined as an ‘infection-permissive state’ in quadrant b (Figure 7A). Similarly, the Clean and *E. coli* O86:B7 groups cluster together and separately from the Mock + *Borrelia*, Live + *Borrelia*, and MAPs + *Borrelia* groups. This clustering identifies them as an ‘infection-refractory state’ in quadrant a (Figure 7A).

To evaluate the impact of *R. helvetica* on the microbial state, the comparison between ticks that fed on infected *R. helvetica* mice but did not become infected (‘infection-refractory state’), and ticks that were found positive to *R. helvetica* infection (‘infection-permissive state’), was performed. The state of the microbiota of infected ticks demonstrated that the infection had an impact on the diversity and the structure of the microbiota, with a drastic decrease in the observed features and of the number of nodes and edges (Figure 7B). On the other hand, ticks that were exposed to *R. helvetica* showed a slight decrease in the observed features, an increase in the number of nodes, and no difference in the number of edges (Figure 7B).

## 4. Discussion

Our results indicate that vaccination with Live bacteria and MAPs led to an increase in the specific IgG levels in the blood of mice, which is consistent with the findings from other studies [18]. Almazan et al. [30] vaccinated mice with SIFamide (SIFa) using a MAP vaccine and allowed the ticks to feed on day 45 after immunization, which is similar to our experimental setup. In their study, an increase in the anti-SIFa IgG was only observed at the time of tick infestation, and later [30]. In our study, an increase in the anti-MAP IgG was observed earlier on the 28th day after immunization (the same day of the third vaccine injection). This difference can be due to different immunogenic properties between peptides [51], or due to the different adjuvant administered [52]. We also observed variability in the IgG response among individual MAP-vaccinated mice. This variability could potentially be attributed to individual differences in immune system function [53].

Regarding antibody cross-reactivity binding, the IgGs from the mice vaccinated with live *S. epidermidis* effectively bound to the proteins extracted from *S. epidermidis*, which is consistent with the findings from previous studies on live bacterial vaccination [18,31,54]. In contrast, the IgGs from the MAP-vaccinated mice did not efficiently recognize the extracted bacterial proteins, indicating a lack of cross-reactivity. This lack of recognition could be explained by multiple factors. One possibility is steric hindrance, where the complex spatial arrangement of the extracted *S. epidermidis* proteins obstructs the antibody access to its epitope [55]. To investigate this issue, assays using the peptide alone could help to determine whether antibodies bind more effectively in a simpler system [55]. Indeed, mice vaccinated with *Staphylococcus* MAPs exhibited increased levels of anti-MAP IgGs, suggesting that steric hindrance may partially contribute to the observed reduction in antibody binding. However, an alternative explanation lies in the nature of peptide-based immune responses. Unlike whole-cell bacterial vaccines, which present a broad array of native epitopes, MAP vaccines rely on a single or limited set of epitopes, leading to a greater variability in immune responses among individuals. This variability is likely due to differences in the antigen presentation and MHC-dependent immune priming, where some individuals may not generate strong responses due to suboptimal MHC binding or inefficient T-cell activation.

The absence of cross-reactivity with native *S. epidermidis* proteins also suggests that the selected IsdB-derived epitope may not be an immunodominant antigen in natural bacterial infections. It is possible that post-translational modifications or conformational differences between the synthetic peptide and the native bacterial protein altered the epitope recognition, preventing effective antibody binding. Additionally, sera from the Live-vaccinated group did not recognize the MAP peptide, which further suggests that the chosen peptide epitope was not a dominant target in the context of a live bacterial infection. In whole-cell vaccines, the immune response is polyclonal, targeting multiple bacterial surface proteins rather than a single epitope. The MAP vaccine, in contrast, relied on a single, defined epitope, which may have been insufficiently exposed or processed differently in vivo, leading to reduced immunogenicity and cross-reactivity [56,57].

Taken together, these findings highlight the following key challenge in peptide-based vaccine design: while synthetic peptides can be effective at eliciting targeted immune responses, their success depends heavily on epitope selection, structural conformation, and the antigen processing efficiency. Future studies should explore alternative *Staphylococcus* surface proteins as vaccine targets, incorporate predictive bioinformatics tools for improved MHC-binding epitope selection, and test different adjuvant formulations to enhance the immune response consistency.

Sera from Live-vaccinated mice exhibited significant bactericidal activity against *S. epidermidis* colonies in vitro, while sera from MAP-vaccinated mice showed a more modest, though still significant, bactericidal effect. Importantly, this effect is independent on the complement system components. This suggest that selecting an immunodominant peptide for immunization could enhance even more the bactericidal effect of produced Abs. These observations suggest a potential link between vaccination and changes in the tick microbiota. Antibodies or immune effectors produced in response to vaccination might influence the composition of the host’s blood, which could, in turn, affect the bacterial communities within the ticks during blood feeding [7]. This modulation of the host’s immune environment may indirectly shape the abundance and diversity of microbial species within the tick microbiome, as seen in our findings with *S. epidermidis*.

Interestingly, vaccination with live bacteria significantly impacted tick fitness by increasing the weight of the ticks. This effect was observed in the ticks that fed on *E. coli*-vaccinated C57BL/6 mice [31,54], but not on those vaccinated with *Leuconostoc* [54]. However, the increased tick weight was not observed in the ticks that fed on *E. coli*-vaccinated alpha-gal knockout (α-gal KO) C57BL/6 mice, where, instead, a high tick mortality was recorded [31]. This highlights the critical role of both the mouse strain and microbial target in shaping outcomes. In the case of *E. coli*, the increase in the tick weight correlated with alterations in the ticks’ microbiota metabolic pathways, which was particularly decreased in the lysine degradation pathway [54]. The observed effect on the tick weight may be attributed to the microbial modulations induced by the vaccine. Ticks fed on mice immunized with live bacteria exhibited a reduction of around 50% in the number of unique taxa when compared to the other two groups. This reduction may indicate a more homogeneous or a less diverse microbial community within the ticks that fed on the live-vaccinated mice. Such a reduction might reflect a loss of certain microbial species or a shift towards a more dominant microbial population in response to the live vaccine, which, in turn, may favor tick fitness. The altered microbiota in the vaccinated mice could influence the tick weight through several mechanisms. For instance, some bacteria play a crucial role in providing the nutrients that are deficient in the host blood, such as vitamins and cofactors, which can directly affect the tick fitness and survival [58]. In addition, a previous study showed that the artificial feeding of *I. ricinus* females with tetracycline-containing blood reduced the levels of Candidatus *Midichloria mitochondrii* in offspring larvae, thus affecting their feeding fitness [59]. Intriguingly, this bacterium was detected in ticks fed on all of the experimental mice groups, and no significant differences in its levels were observed among the groups. Additional studies are warranted to determine how the microbiota dysbiosis caused by mice immunization with live *S. epidermidis* enhances tick feeding.

Ticks fed more rapidly on mice vaccinated with the live vaccine and more slowly on mice vaccinated with MAPs compared to the control group. This difference in engorgement might suggest that the immunization with the MAPs alters the tick fitness, consequently reducing the optimal blood feeding rates. On the other side, a longer feeding period may favor pathogen transmission. For example, the transmission of *B. burgdorferi* is higher with prolonged feeding, as the ticks remain attached for longer periods [58]. No effect of any *Staphylococcus* vaccine (live or MAPs) on the tick mortality rates was observed in our study. In contrast, Wu-Chuang et al. [18] reported a significant decrease in the mortality rate of ticks fed on *E. coli*-vaccinated wild-type mice, while Mateo-Hernandez et al. [31] demonstrated an increase in the mortality for ticks that fed on *E. coli*-vaccinated α-gal KO C57BL/6 mice, which suggests a taxon-specific modulation between *S. epidermidis* and *E. coli* vaccination, and a mice strain-specific modulation of the tick fitness.

Analysis of the tick microbiota revealed no significant differences in the alpha diversity among the different groups, suggesting that the overall richness and evenness of microbiota within individual ticks were similar across groups [60]. However, while the beta diversity of the mock-immunized group remained consistent with that of the live and MAP-immunized groups, we observed a significant shift in the beta diversity between the ticks immunized with MAPs and those immunized with live bacteria. This result indicates that a distinct vaccination formulation differentially impacts the composition of the tick microbiota [60].

Microbial network analysis highlighted the significant role of the *Morganella* taxa in the Mock and MAP groups. *Morganella*, a Gram-negative bacterium from the order Enterobacterales, includes *M. morganii*, which can act as an occasional human pathogen [61]. In the MAP group, *Morganella* is central to the overall microbiota structure, and the robustness test predicted that the bacterium with the highest betweenness centrality score was associated with a substantial loss of network connectivity if removed. Previous studies have documented the presence of *Morganella* in the microbiota of *I. ricinus* in the Czech Republic [62] and *Haemaphysalis longicornis* in China [63]. The importance of *Morganella* in our study underscores its potential role in maintaining the microbial community structure, especially in the context of MAP immunization.

Murine strains exhibit low susceptibility to infection by bacteria of the genus *Rickettsia* [64]. Among these, the C3H/HeN strain is known to be susceptible to *Rickettsia*, making it a widely used model for studies on host–pathogen interactions [65,66,67,68]. In a previous study, we successfully infected C3H/HeN mice with the *R. helvetica* strain DK2, marking the first establishment of an animal model for this bacterium [19]. Although over 89% of the mice were positively infected, only about 6% of *Ixodes ricinus* nymphs acquired *R. helvetica*. Similarly, a low infection prevalence was observed in other tick species, such as *Amblyomma sculptum*, the primary vector of *Rickettsia rickettsii* in Brazil [69,70]. Additionally, wild ticks also exhibited a low prevalence of *R. helvetica* infection [71,72]. Although the presence of *Staphylococcus* correlates with the absence of *R. helvetica* in wild *I. ricinus* [13], *Staphylococcus*-based vaccines did not enhance rickettsial acquisition by ticks.

The higher infection rate of *R. rickettsii* in *A. aureolatum* compared to *A. sculptum* was correlated with a more furnished microbiota predominantly composed of *Francisella* in *A. aureolatum,* compared to a sparser microbiota in *A. sculptum,* demonstrating that the microbiota abundance impacts the pathogen transmission rate [73]. For *Borrelia* infection, our results demonstrate that the ticks with a less diverse microbiota are more susceptible to infection than those with a highly diverse one. However, the modulation of the tick microbiota with an *E. coli* antimicrobiota vaccine reduced the microbial diversity and was associated with significantly lower spirochete numbers than in the control group [18]. This suggests that the relationship between microbial diversity and pathogen susceptibility is complex, and may depend on specific microbial interactions or immune responses induced by the vaccine. Conversely, vaccination with *S. epidermidis* did not reduce the microbial diversity but increased the microbial connectivity compared to the control. This result suggests a taxon-specific modulation of vaccines on the microbiota modulation, as previously observed [54]. Therefore, the selection of the bacterial target is crucial for the effects on ticks. The ability for a vector to transmit pathogens depends on its microbial structure [7]. Targeting keystone taxa has proven to have a greater impact on the microbiota than vaccination with random taxa [54]. Our data indicate that microbiota modulation influences the tick microbiota connectivity and structure in a taxon-specific manner. While *Staphylococcus*-based antimicrobiota vaccination altered the microbiota network without affecting the pathogen acquisition, *E. coli*-based vaccination induced a microbial state associated with reduced *Borrelia* colonization (Figure 7). These findings suggest that the taxonomic composition of microbiota modifications, rather than microbiota alteration per se, determines whether a pathogen-permissive or pathogen-refractory state is achieved.

Although the microbial composition and connectivity are influenced by various factors, including the targeted pathogen, tick species, and the origin of the ticks (lab-reared vs. wild), our findings suggest that microbiota-targeted vaccines have the potential to induce a state of microbial refractoriness to pathogen acquisition. However, this effect may be taxon-dependent, as suggested by differences in the microbiota modulation observed in this study and prior research on *E. coli*-based modulation [18]. Our findings, based on reanalysis of previously published data [18] and our own experimental results, show that, while *E. coli*-based microbiota modulation is associated with reduced *Borrelia* colonization [18], *Staphylococcus*-based modulation primarily altered the microbiota connectivity (Figure 7) without significantly affecting the pathogen acquisition. This suggests that microbiota modifications must reach a specific structural or functional threshold to confer pathogen refractoriness, which varies depending on the microbial taxa involved. However, direct experimental comparisons across multiple taxa are necessary to confirm this hypothesis. This approach could potentially enhance the control of tick-borne diseases by leveraging microbiota modulation as a strategy to reduce the pathogen infection in ticks. One limitation of this approach is that the outcome of the vaccination cannot be predicted with certainty before the administration. In the current study, despite *Staphyloccous* being highly abundant in *I. ricinus* [13], and the found keystone taxa in wild *Rhipicephalus bursa* ticks [74], its vaccination is not effective against *B. afzelii* pathogen. Only empirical methods can definitely determine the success or failure of the antimicrobiota vaccine’s effect on tick fitness and vectorial capacity.

## 5. Conclusions

Our findings show that antimicrobiota vaccines influence the tick microbiota and fitness, but their effects on pathogen acquisition appear to be contingent on both taxon-specific differences and peptide selection. The distinct outcomes of live *S. epidermidis* and MAP-based vaccines highlight the importance of both the bacterial taxon and epitope choice in designing effective microbiota-targeted interventions.

While *E. coli*-based microbiota modulation has been linked to pathogen refractoriness, Staphylococcus-based vaccination altered the microbiota connectivity without reducing the pathogen acquisition. This suggests that effective microbiota-targeted vaccines require selecting both the right bacterial species and the right immunogenic peptide. Future research should identify bacterial taxa whose microbiota modulation correlates with reduced pathogen acquisition and refine the epitope selection within those species.

Additionally, optimizing bioinformatics-driven epitope prediction, MHC-binding selection, and adjuvant formulations may improve the vaccine efficacy. By integrating bacterial taxon selection with targeted peptide design, microbiota-targeted vaccines may be better optimized for vector control and disease prevention.

## Figures and Tables

**Table 1:** Topological features of the microbial networks.

Network Parameters	Mock Group	MAPs Group	Live Group
Nodes	13	25	25
Edges (% positive)	11 (100)	29 (97)	19 (100)
Average degree	1.69	2.32	1.52
Average Path Length	1.74	3.94	1.17
Diameter	4	8	2
Clustering Coefficient	0.24	0.41	0.76
Modularity	0.63	0.71	0.81
Number of Modules	3	5	10

## Data Availability

The data presented in this study are openly available in SRA, reference number bioproject Nos. PRJNA1145395; PRJNA1065249, and PRJNA803003.

## Supplementary Materials

The following supporting information can be downloaded at: https://www.mdpi.com/article/10.3390/pathogens14030206/s1, Figure S1. Serological detection of anti-Borrelia IgGs; Figure S2. Detection of *R. helvetica* infection; Table S1. Microbial taxa distribution across groups.
