# Supplementary figures and images for: Effects of Live and Peptide-Based Antimicrobiota Vaccines on Ixodes ricinus Fitness, Microbiota, and Acquisition of Tick-Borne Pathogens
Source: Pathogens. 2025 Feb 20;14(3):206. doi: 10.3390/pathogens14030206 (PMC11945021; doi:10.3390/pathogens14030206)

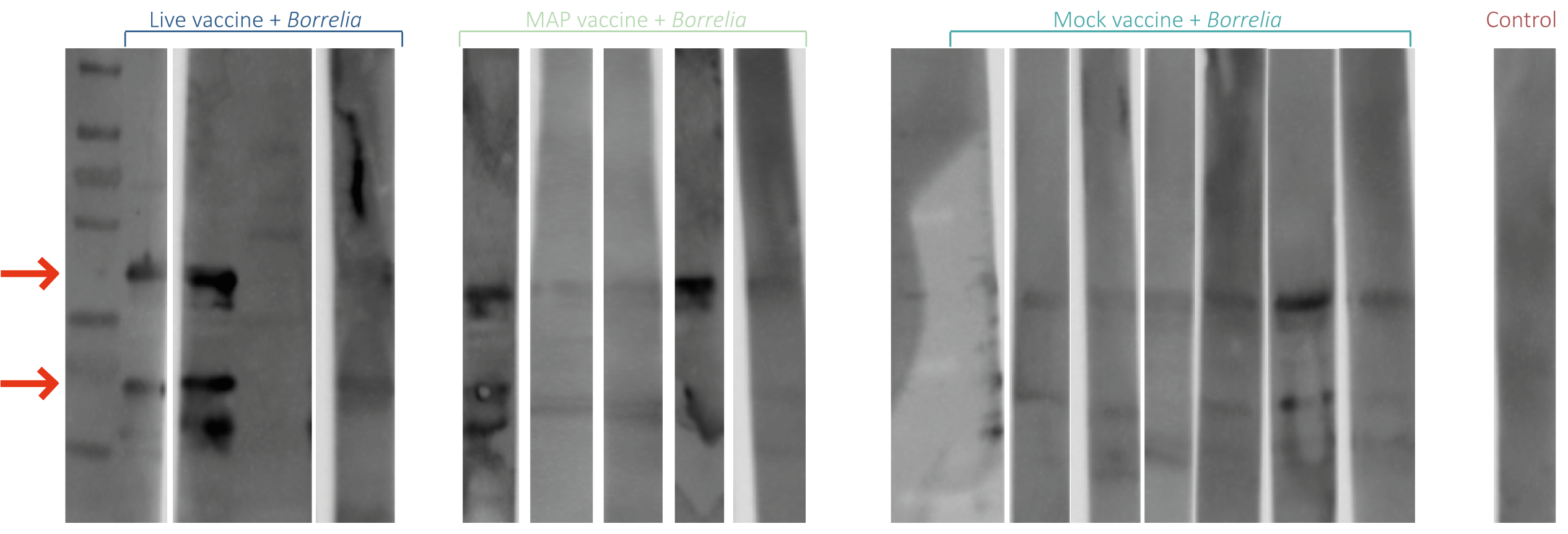

Supplement: Supplementary file 1 [file pathogens-14-00206-s001.zip › Supplementary files R3/Supplementary figure 1.tif]

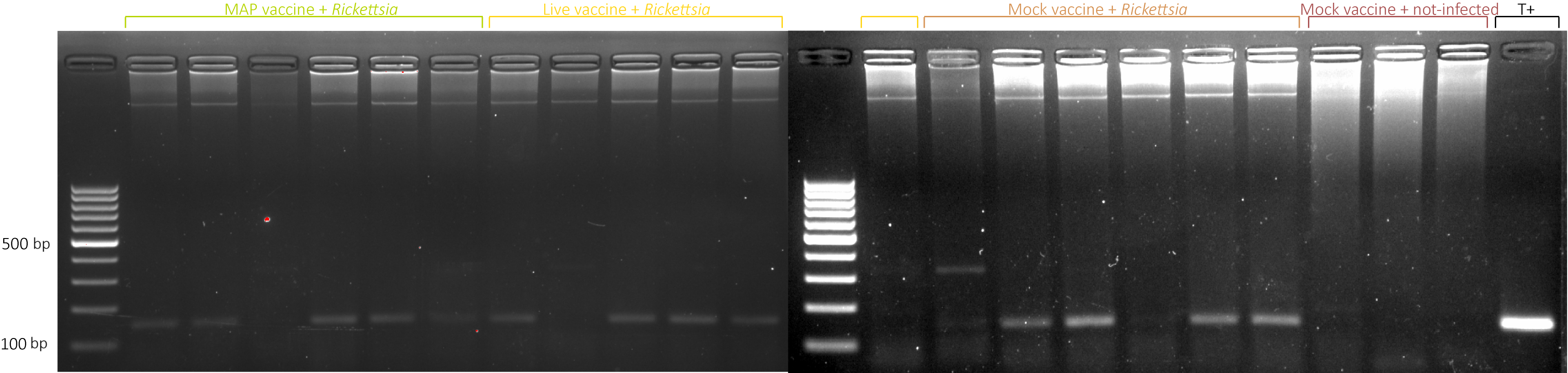

Supplement: Supplementary file 1 [file pathogens-14-00206-s001.zip › Supplementary files R3/Supplementary figure 2.tif]
